# Maternal Dystokia1Read at the annual meeting of the Veterinary Medical Association of New Jersey.

**Published:** 1901-08

**Authors:** T. Earle Budd

**Affiliations:** Orange, N. J.


### MATERNAL DYSTOKIA.[1]

By T. Earle Budd,

ORANGE, N. J.

Under the head of "Maternal Dystokia" I want to speak of the one form which has caused me the most anxiety and most labor, and which you will all recognize at once—"Rigidity of the Cervix Uteri." This condition is more frequently met with in the cow than in other animals. Next to the cow I would say the mare. I have also found it more frequent in well-bred, well-cared for, and well-fed animals. Without modifications in the structure of the cervix, but merely by a kind of rigid constriction of its fibres, the os remains closed, and the more the animal strains the more dense it seems. Some of our writers claim it to be a spasm. From my own experience I would say it is simply a passive condition of the cervix. The symptoms do not vary, the pains come and go as if everything was normal, and this condition will in a majority of cases continue for ten or twelve hours, when the owner will either try himself or send for a veterinarian.

It may be the labor pains will seem to disappear for a time, and then return; now is the time we are sure to be sent for. Now,

[1] Read at the annual meeting of the Veterinary Medical Association of New Jersey.

with an ofttimes confused history, we make an examination, and our faces grow long and our hearts sink within us, for if the exploration has been carefully made the state of the cervix will at once explain the cause of the delay in the delivery. When the hand comes in contact with the cervix, which you will not find at once, you are disappointed, for you who have had experience know what is before you, and you who dimly recollect it from your lecture-room are somewhat puzzled. However, we all recognize the condition, even if it is our first experience.

The treatment should be slow. Do not expect to accomplish much in the first five or ten hours. First explain to the owner the conditions and your idea of the prognosis, then make yourself as comfortable as possible. This consists in rubber boots, overalls, and blouse without sleeves. Then the animal and surroundings: Have the stall clean, roomy, and well littered with clean straw. Now give the cow or mare one ounce of chloral. Do not undertake this without proper attendants. I would suggest two at least. Have one hold the animal by the head. After preparing the arm with carbolized oil, enter the hand and carefully find the cervix. You find you cannot enter it, not even with the finger. You must not be discouraged. Place either the first or second finger directly over the cervix, and rotate the hand slowly; continue this for some time. After from thirty to forty-five minutes, if you find you have made no progress, try a small sponge wet with the fluid extract of belladonna. If you are successful in getting the sponge between the external and the internal rings, let it remain overnight. When you return in the morning do not be disappointed if you cannot accomplish your task, for I must say of the many cases it has been my misfortune to have I have yet to see the first case where I have realized any good from the sponge and belladonna. I think we all would save time and shorten our period of anxiety if we would apply irrigation. Now is the time you will need your two assistants, for this, you will find, will be quite an undertaking. First of all, do not commence until you are ready; tell your assistants just what they are expected to do; have two or three barrels of water as near 105° F. as possible. This, however, you must regulate, as the temperature of the weather will necessarily affect the temperature of the water. With six or seven feet of small rubber hose and a good-sized funnel at one end, I carry the other end directly to the cervix; then have my assistant stand as high as possible and keep the funnel filled. The reason I use a long and small hose is, first, I want a continual stream; second, if she should resent it, I will not only save a good drenching, but the stream will not cease. After you have used say one barrel of water, without removing the hose, make an examination with the finger, and if you find the external ring has dilated, even a little, then stop irrigation; oil the hand; try to pass it through, but go slowly. If you cannot, after finding the external ring somewhat dilated, let the animal rest for five or six hours. When you return in all probability you can deliver the fœtus. I am now speaking of the cow. With the mare you must work much faster. As I have only had one case with the mare, and came near losing mare and fœtus by waiting, I would advise, from my limited experience, to work rapidly. In all cases stimulate the mother. I think our instructors in college taught us to operate. I have had some conversation with two professors of obstetrics, and asked what the prognosis would be, and they each said death. Why I insist on going slow in the cow is from good results obtained by myself by so doing.

I will relate one particular case, showing it paid me. On February 14, 1898, in the evening, I was called to see a very valuable cow, the owner having refused $200 for her, and from my judgment he was wise. When I arrived I found this cow had been in labor some ten or twelve hours. I at once made an examination and found I had trouble. After exhausting all other means, on the 16th I used the irrigation at eleven o'clock at night. I was able to pass my hand through both rings, and to my surprise I found the fœtus alive, and by 11.30 I had accomplished what seemed to me at first impossible. This calf was one of the few weighing over 100 pounds.

It paid me to go slow.

---

LAW TO REGULATE THE PRACTICE OF VETERINARY MEDICINE IN FRANCE. The most salient points in the law enacted in January last are: 1. No one is allowed to practice unless he holds a diploma from one of the French national veterinary schools, excepting those non-graduates who have been licensed to practice for a period of at least three years before the passage of the act. 2. The law does not apply to castrators. 3. Foreign graduates are not allowed to practice without first obtaining a diploma from one of the French schools. 4. Veterinarians cannot be proprietors of drug stores; they are authorized only to prepare and deliver medicines for animals confined to their care.—*Rec. de Méd. Vét.*

